# Impact of long-term treatment of onchocerciasis with ivermectin in Ecuador: potential for elimination of infection

**DOI:** 10.1186/1741-7015-5-9

**Published:** 2007-05-23

**Authors:** Juan Carlos Vieira, Philip J Cooper, Raquel Lovato, Tamara Mancero, Jorge Rivera, Roberto Proaño, Andrea A López, Ronald H Guderian, José Rumbea Guzmán

**Affiliations:** 1 Programa Nacional de Eliminación de la Oncocercosis en el Ecuador, Servicio Nacional de Control de Enfermedades Transmitidas por Vectores Artrópodos, Ministry of Public Health, Guayaquil, Ecuador; 2 Desarrollo Comunitario Vozandes HCJB, Casilla 17-17-691, Quito, Ecuador; 3 Pan American Health Organization, Georgetown, Guyana

## Abstract

**Background:**

Onchocerciasis is a leading cause of blindness worldwide, hence elimination of the infection is an important health priority. Community-based treatment programs with ivermectin form the basis of control programs for the disease in Latin America. The long-term administration of ivermectin could eliminate *Onchocerca volvulus *infection from endemic areas in Latin America.

**Methods:**

A strategy of annual to twice-annual treatments with ivermectin has been used for onchocerciasis in endemic communities in Ecuador for up to 14 years. The impact of ivermectin treatment on ocular morbidity, and *O. volvulus *infection and transmission was monitored in seven sentinel communities.

**Results:**

Over the period 1990–2003, high rates of treatment coverage of the eligible population were maintained in endemic communities (mean 85.2% per treatment round). Ivermectin reduced the prevalence of anterior segment disease of the eye to 0% in sentinel communities and had a major impact on the prevalence and transmission of infection, with possible elimination of infection in some foci.

**Conclusion:**

The distribution of ivermectin in endemic communities in Ecuador might have eliminated ocular morbidity and significant progress has been made towards elimination of the infection. A strategy of more frequent treatments with ivermectin may be required in communities where the infection persists to achieve the objective of elimination of the infection from Ecuador. The elimination of the infection from an endemic country in Latin America would be a major public health achievement and could stimulate the implementation of elimination strategies in other endemic countries.

## Background

Onchocerciasis is an important blindness-causing disease resulting from infection by the filarial parasite *Onchocerca volvulus*, transmitted by *Simulium *blackflies. An estimated 18 million persons are infected worldwide [1], and approximately 770000 are blind or visually impaired as a consequence of the infection [1]. The infection also causes severe and troublesome skin disease [2] and is associated directly with increased mortality [3].

Ivermectin (Mectizan^®^) is an oral microfilaricidal drug that is safe and effective for mass treatment of onchocerciasis [4] and is provided free to endemic countries by Merck & Co. The Onchocerciasis Elimination Program for the Americas (OEPA) has coordinated the distribution of ivermectin since 1991 in the six endemic countries in Latin America (Brazil, Colombia, Ecuador, Guatemala, Mexico and Venezuela) with two principal goals: to eliminate morbidity caused by onchocerciasis by 2007 and to eliminate the infection where possible using a strategy of mass treatment of endemic communities with ivermectin [5].

Elimination is a high priority to prevent new morbidity, to allow the reallocation of limited health and financial resources to other health priorities, and because of the potential for the development of parasite resistance to ivermectin in the long term [6]. The development of resistance to ivermectin by *O. volvulus *is a serious concern because there are no safe or practical alternative treatments currently available for mass distribution in human populations. It has been suggested that the infection could be eliminated from the Americas, through twice-annual treatments with ivermectin to greater than 85% of the eligible population at risk for onchocerciasis over a period of 10–15 years, via the interruption of the transmission of infection and death of adult parasites by senescence [7].

In Ecuador, the National Program for the Elimination of Onchocerciasis has pursued a program of mass treatments with ivermectin in endemic communities over the past 14 years (1990–2003) and has achieved high rates of coverage. The Ecuadorian Program provides an important model to evaluate whether elimination of *O. volvulus *infection is a realistic aim for an endemic country in the Americas using an ivermectin-based treatment strategy.

## Methods

### Study area

**Figure 1:**
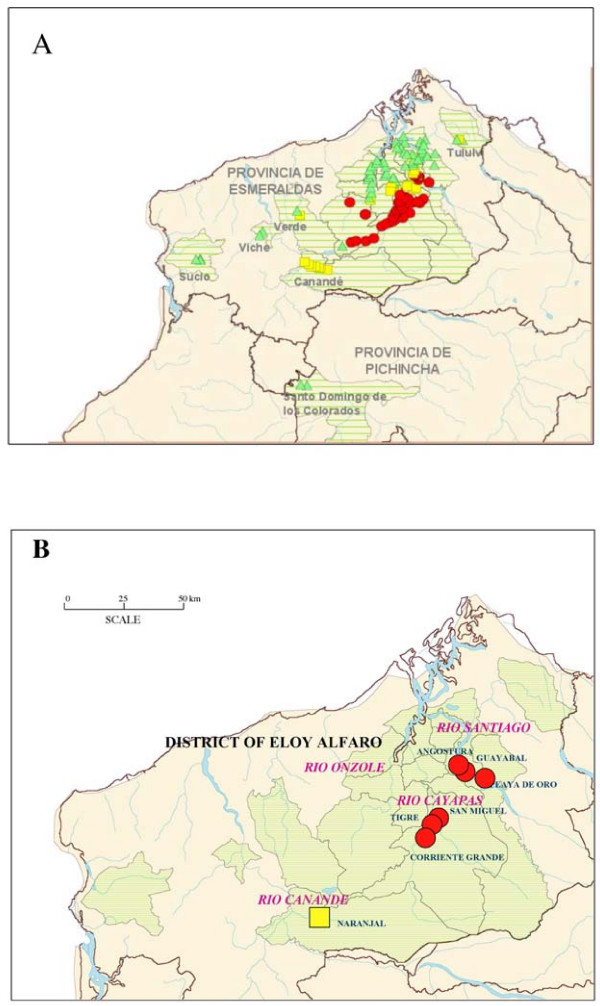
**Geographic locations**. (A) Geographic location of endemic area for onchocerciasis in Ecuador. The endemic focus is located in the Northern coastal area of Ecuador adjacent to Colombia. There are a total of 119 endemic communities for onchocerciasis located in Esmeraldas Province (117 communities) and Pichincha Province (two communities). The endemic area consists of hyperendemic (red circles), mesoendemic (yellow squares), and hypoendemic (green triangles) communities. There are six satellite foci (Tuluví, Verde, Viche, Canandé, Sucio and Santo Domingo de Los Colorados). The areas in green represent the foci of onchocerciasis. (B) Geographic location of the seven sentinel communities in the District of Eloy Alfaro in Esmeraldas Province. Hyperendemic (red circles), and mesoendemic (yellow square) sentinel communities are shown. The areas in green represent the foci of onchocerciasis.

The province of Esmeraldas, latitude 1°96'S to 1°43'N and longitude 78°48'W to 80°9'W, is situated west of the Andes in North-Western Ecuador [8]. The principal endemic area of onchocerciasis is located in the District of Eloy Alfaro (Figure 1). The Santiago basin focus is formed by the union of three rivers, the Río Santiago, Río Cayapas and Río Onzole and their respective tributaries, each forming a separate sub-focus. Satellite endemic foci are located on separate rivers caused by the migration of microfilariae-positive individuals from the principal endemic focus [9]. Five satellite foci are found in the province of Esmeraldas, located on the rivers Río Canandé, Río Verde, Río Viche, Río Sucio, and Río Tuluví. A small focus is also found in the neighboring province of Pichincha near Santo Domingo de los Colorados. A total of 119 communities are considered endemic for onchocerciasis.

### Ivermectin distribution

The National Program for Onchocerciasis Elimination in Ecuador chose to use ivermectin as the disease control strategy through mass drug distribution in endemic communities since 1990. Before 1990, control relied largely on periodic nodulectomy campaigns in the endemic areas [10]. The distribution of ivermectin was started in endemic communities over the period 1990 to 1993 although treatment was delayed in a few until 1997. All consenting eligible inhabitants (approximately 19420) of the 119 communities have been treated for up to 14 years (1990–2003). Eligibility criteria for treatment were: weight greater than 15 kg, women not pregnant or nursing infants up to 3 months of age, and free of serious illness (e.g. active tuberculosis, terminal cancer etc). Ivermectin is distributed by specially-trained primary health care workers in each community at a dose of 150 μg/mg using standard exclusion criteria [11]. The population census is used as the basis for drug distribution. Health workers are responsible for health education, updating the census annually, identifying those not eligible for treatment and treating all eligible individuals.

Ivermectin has been distributed annually to endemic communities in the Río Cayapas since 1991–1992 except for seven communities in the hyperendemic area of the Río Cayapas sub-focus that received their first mass treatment between 1995 and 1996. A second annual dose of ivermectin was provided to hyperendemic communities in the Río Cayapas over the periods 1992–1994 and 1998–2003. A second dose was not given in the years 1995–1997 due to financial restraints on the program and a decision to fall in line with the strategy of annual treatments adopted by the African Program for Onchocerciasis Control (APOC) [7]. However, following the finding that twice-annual treatments could suppress onchocerciasis transmission in the Santiago sub-focus [11], the strategy of twice-annual treatments in hyperendemic communities was reinitiated from 1998. All endemic communities have been treated twice annually since 2001. The number of treatments and the start of ivermectin mass distribution provided to each river sub-focus and the satellite foci according to endemicity of infection are provided in Table 1.

### Sentinel communities

The use of sentinel communities has been recommended for the in-depth evaluation of ivermectin distribution programs [1]. To evaluate the impact of mass distribution of ivermectin, seven communities were selected for periodic prospective in-depth epidemiological surveys. The seven sentinel communities were located in the following areas of the Santiago basin main focus: (1) hyperendemic area – Río Santiago: Playa del Oro, Angostura and Guayabal communities; (2) hyperendemic area – Río Cayapas: Corriente Grande, El Tigre and San Miguel communities; and (3) mesoendemic area – satellite focus in Río Canandé: Naranjal de los Chachis community. Sentinel communities in the principal hyper-endemic sub-foci of the Río Santiago and Río Cayapas were chosen to evaluate the potential for reduction in transmission of infection through ivermectin treatment. A meso-endemic community in the Río Canandé was included because of large changes in prevalence that occurred over the period 1980–1986 and the high infectivity and human bite rates in this satellite focus [9]. Evaluations were performed before the start of ivermectin treatment in 1989 and in 2000 and 2004. The period from the last treatment with ivermectin provided in 2003 to the evaluations performed in 2004 was approximately 12 months. Parasitological ophthalmological, and entomological parameters were evaluated using standardized methodology [12]. Selection for inclusion in the parasitological and opthalmological assessments were: (1) parasitological – all individuals in the community census aged 1 year or greater, and (2) ophthalmological – all individuals in the census aged 10 years or greater. The study was approved by the Ethics Committee of the Hospital Vozandes, Quito, Ecuador. Informed verbal consent to participate in the study was obtained through meetings of community elders and the individual participants.

### Prevalence of infection and microfilarial load

Skin snips were taken from both iliac crests using a Stolz corneoscleral punch and weighed. The biopsies were placed in micro-well plates with phosphate buffered saline, and emergent microfilariae were counted microscopically after 24 hours. Microfilarial densities are expressed as the number of microfilariae per milligram of skin (mf/mg). Mean microfilarial load (MMFL) was calculated as the geometric mean of infection intensities of all members of the community including skin-snip negatives (i.e. e^Sum(loge[x+1])/N-1^, where x is individual microfilarial loads and N is the number of individuals examined).

### Ophthalmology

Ocular examinations included assessment of visual acuity, slit lamp examination of the anterior segment, and direct and indirect ophthalmoscopy of the posterior segment [13]. All examinations were performed by two experienced ophthalmologists (JR and RP) using the modified rapid ocular evaluation criteria recommended by the Pan American Health Organization [14].

### Entomology

*Simulium *blackflies were collected in the seven sentinel communities during the seasonal period of maximum biting in 1995 in the sentinel communities of Tigre and San Miguel and in all sentinel communities in 2000 and 2004. Standard procedures were followed for blackfly collections [15]. Blackflies were collected in 99% alcohol and identified using a taxonomic key of *Simulium *[16]. *O. volvulus *DNA was amplified by PCR using primers for the *O. volvulus*-specific DNA sequence O-150 [17] in pools of 50 blackflies, and the *O. volvulus*-specific PCR products were detected by ELISA as reported previously [17]. The prevalence of infection was calculated using the algorithms contained in Poolscreen 2.0 (The University of Alabama, Birmingham, Alabama, USA), a statistical program that estimates the prevalence of infection in the vector population based upon the proportion of positive pools [18].

## Results

### Ivermectin treatments and treatment coverage in all endemic communities

The average annual treatment coverage per treatment round with ivermectin from 1990 to 2003 (total of up to 24 treatments) of eligible individuals in endemic communities receiving treatment, based on a yearly updated census, was 85.2% (range 54.9–97.9%). Between 1990 and 2000, annual mass distribution of ivermectin was provided in endemic communities except for seven communities in the Río Cayapas where treatment was delayed until 1995–1996 as described in the Methods section. From 2001, all 119 endemic communities were treated twice annually with ivermectin. Between 1990 and the end of 2003, prior to the evaluations in 2004, endemic communities received a range of 11–22 total treatments with ivermectin (Table 1). Average coverage per treatment round of ivermectin was high (>83%) in all endemic foci (Table 1).

### Characteristics of sentinel communities

The population eligible for treatment with ivermectin, number of mass treatments, treatment strategy by year (single vs. twice annual), and treatment coverage are shown in Table 2. The age and sex distribution of the sentinel communities were similar between the three surveys. The number of treatment rounds was not uniform for all study communities. Corriente Grande, Río Cayapas, received a total of 21 ivermectin treatments while El Tigre and San Miguel on the same river received 13 treatments. The average treatment coverage was ≥ 85% except for Guayabal (Río Santiago) and Naranjal (Río Canandé).

### Impact of ivermectin: *O. volvulus *infection prevalence and MMFL

The number of subjects aged 1 year or greater in the 2004 census in each sentinel community and the proportions examined (brackets) in the 2004 survey were: Corriente Grande, 212 (83.0%); El Tigre, 143 (67.8%); San Miguel, 197 (79.2%); Playa de Oro, 267 (71.9%); Guayabal, 114 (65.8%); Angostura, 83 (67.5%); and Naranjal, 581 (83.0%). Overall, 77.3% of these 1597 individuals were examined. Marked reductions of prevalence of infection were seen in the sentinel communities over the periods of mass distribution of ivermectin (Table 3). In the 2004 survey, all persons examined in four of the sentinel communities were negative for detectable microfilariae in skin snips, but positive skin snips were detected in all the sentinel communities in the hyperendemic area in the Río Cayapas where 5 individuals had positive skin snips (Corriente Grande, 1 individual; El Tigre, 2; and San Miguel, 2). Infected individuals had a median age of 46 years (range 27–49 years) and had received a median of 11 ivermectin treatments (range 6–13 treatments). All but one had onchocercal nodules. A further four skin-snip negative individuals (median age 15 years [range 8–21] and median 14 treatments [range 4–17]) developed localized maculopapular rashes approximately 24 hours after ivermectin treatment given immediately after the clinical evaluations. No children aged 5 years or less were positive for microfilariae in skin snips. There were reductions in the microfilariae densities in all sentinel communities over the treatment periods (Table 3).

### Impact of ivermectin: anterior segment eye lesions

The number of subjects aged 10 years or more (i.e. able to cooperate fully with the ocular examination) in the 2004 census in each sentinel community and the proportions examined (brackets) in the 2004 survey were: Corriente Grande, 132 (93.9%); El Tigre, 87 (83.9%); San Miguel, 129 (86.0%); Playa de Oro, 198 (68.2%); Guayabal, 78 (66.7%); Angostura, 55 (78.2%); and Naranjal, 387 (81.7%). Overall, 80.1% of these 1066 individuals were examined. Data is provided only for the early anterior segment changes (punctate keratitis and the presence of microfilariae in the anterior chamber) that are considered to be specific for *O. volvulus *infection, resolve completely after effective chemotherapy and provide a strong indication for reduction in ocular morbidity and the risk of later severe ocular disease (e.g. iridocyclitis, sclerosing keratitis, chorioretinopathy and optic atrophy) after ivermectin treatment. The prevalence of punctate keratitis before treatment and in the surveys conducted in 2000 and 2004 is shown in Table 4. By 2000, there were reductions in the prevalence of punctate keratitis in all sentinel communities and no evidence of punctate keratitis was found in Angostura in the Río Santiago. No evidence of punctate keratitis was observed in the 2004 survey in any of the sentinel communities. No microfilariae in the anterior chamber were observed in any of the sentinel communities in the 2000 and 2004 surveys.

### Impact of ivermectin: transmission of *O. volvulus *infection

All anthropophilic biting blackflies in the seven sentinel communities were identified as *S. exiguum *or *S. quadrivittatum*. The predominant species collected in the sentinel communities in the Río Santiago was *S. quadrivittatum *(22.1% *S. exiguum *vs. 77.9% *S. quadrivittatum *in 2000; 11.5% *S. exiguum *vs. 88.5% *S. quadrivittatum *in 2004), in contrast to the Río Cayapas where *S. exiguum *predominated (76.2% *S. exiguum *vs. 23.8% *S. quadrivittatum *in 2000; 78.5% *S. exiguum *vs. 21.5% *S. quadrivittatum *in 2004). In Naranjal in the Río Canandé, *S. exiguum *was the principal human biting species (2000, 99.0% *S. exiguum *vs. 1.0% *S. quadrivittatum *in 2000; 99.2% *S. exiguum *vs. 0.8% *S. quadrivittatum *in 2004). The blackflies were analyzed for the presence of *O. volvulus *DNA (O-150) by PCR-ELISA (2009 pools analyzed for 2000 and 1845 pools for 2004). Infection rates per 10000 flies are shown in Table 5. No evidence of infection of *Simulium *blackflies was detected in the three sentinel communities in the Río Santiago in 2000 and 2004. In the communities of Corriente Grande (Río Cayapas) and Naranjal (Río Canandé), blackfly infection rates declined to 0 over the period 2000–2004. However, blackfly infections were detected during both surveys in the communities of El Tigre and San Miguel in the Río Cayapas. *S. exiguum *appeared to be the most important vector during both surveys and *O. volvulus *infection in *S. quadrivittatum *was detected only in Corriente Grande in 2000 but in no other sentinel community in 2000 or 2004.

## Discussion

The National Program for Onchocerciasis Elimination in Ecuador has used the phased introduction of mass distribution of ivermectin in endemic communities to control onchocerciasis as a public health problem since 1990. The findings of the in-depth surveys conducted in 2000 and 2004 provide some evidence that the infection has been eliminated in the sentinel communities in the Río Santiago and Río Canandé. Because of the high coverage rates achieved, it is possible that *O. volvulus *infection has been eliminated from the Río Santiago sub-focus and all satellite foci including the Río Canandé sub-focus. However, *O. volvulus *infection is still active in the hyperendemic sub-focus of the Río Cayapas as demonstrated by the presence of individuals with dermal microfilariae and infected *Simulium *blackflies.

Unlike foci of onchocerciasis in Africa, the foci in the Americas are geographically well defined and are amenable to focused attack that, theoretically, could lead to elimination of the infection from the continent. With this in mind, The PanAmerican Health Organization in 1991 called for the elimination of all morbidity from onchocerciasis in the Americas and where possible suppression of transmission by 2007 [5,7].

The rationale for the current ivermectin control strategy is derived from studies conducted in Guatemala that indicated that twice-annual distribution of ivermectin to all eligible persons could suppress transmission [19,20], and the results of simulation studies with a mathematical model that indicated that suppression of transmission could be achieved by treatment of at least 85% of the eligible population [7]. If suppression of transmission can be maintained in an isolated focus for the reproductive life span of adult females, estimated at 13–14 years [21], the adult parasite population would die by senescence [22] and the infection would be eliminated.

We have demonstrated previously that an operational program based on ivermectin distribution twice annually over a period of 5 years in the Río Santiago sub-focus appeared to completely suppress *O. volvulus *transmission [11]. We have now extended these findings to show that transmission of *O. volvulus *in the Río Santiago might have been interrupted for at least 10 years (1995–2004). There is no evidence of new onchocercal ocular morbidity in the Río Santiago and dermal microfilariae have been absent in the three sentinel communities since 1996 [11]. Further, studies to detect IgG4 antibodies against the *O. volvulus *antigen OV16 using immunochromatic cards that are highly sensitive and specific for *O. volvulus *infection [23] were negative in 112 children aged less than 11 years in 2001 (i.e. children born since 1990 when the first dose of ivermectin was administered; Rumbea Guzman et al, unpublished data) providing further evidence for an interruption of transmission. These data together indicate that *O. volvulus *infection, after at least 13 years of continuous annual or twice-annual mass treatments with ivermectin, has now been eliminated from the Río Santiago sub-focus.

*O. volvulus *infection remains active in the hyperendemic communities in the Río Cayapas. Individuals with dermal microfilariae were detected in three sentinel communities (total of 5 individuals) during the 2004 survey and infected blackflies were detected in two sentinel communities. Two of the three communities had received fewer ivermectin treatments than other sentinel communities having started to receive treatment in 1996 (Table 2). Microfilariae positive individuals were aged greater than 25 years and tended to have evidence of onchocercal nodules (4/5 individuals). The use of skin snips to detect *O. volvulus *infection in humans is relatively insensitive and it is likely that there is a larger reservoir of active infection in these communities than suggested by the skin snip results. The latter is supported by the finding of a further four individuals in the hyperendemic sentinel communities in the Río Cayapas that had negative skin tests for microfilariae but probable microfilaricidal skin reactions after ivermectin at the time of the 2004 survey. It is not clear how active is infection transmission because a serological study using the OV16 immunochromatic cards conducted in 2001 showed 2.1% (3/145) of subjects aged 0–5 years to have evidence of specific antibodies (Rumbea Guzman et al, unpublished data) although no children in this age range had positive skin snips for microfilariae. Although infection remains present, there was no evidence of new onchocercal ocular lesions in any of the sentinel communities in the Río Cayapas indicating that ocular onchocerciasis might have been eliminated as a public health problem.

All of the hyperendemic communities in the Río Cayapas have received at least 5 years of twice-annual treatments with ivermectin (Table 1) and the finding of active infection is in contrast to our previous findings of the possible complete suppression of infection in the Río Santiago after 5 years of twice-annual treatments [11] indicating possible differences in the minimum effective duration of ivermectin mass treatments required to suppress transmission between the two infection sub-foci. There are three possible explanations for this difference: (1) the predominant vector in the Río Cayapas sentinel communities, *S. exiguum*, has a higher vectorial capacity than *S. quadrivittatum *that predominates in the Río Santiago [24,25]; (2) the same infected individuals might refuse treatment repeatedly, or are ineligible for long periods (e.g. through repeated pregnancies), and remain an active source of transmission of *O. volvulus *or temporary migrations of infected individuals in and out the Río Cayapas and the failure of them to receive repeated treatments could maintain the infection reservoir (this appears an unlikely explanation as all these individuals found positive had received significant numbers of ivermectin treatments – 10/13, 6/13, 12/13, 13/13, and 11/21); and (3) a sub-population of adult female *O. volvulus *worms appear to respond poorly to the fertility suppressant effects of ivermectin, and this effect is observed after repeated doses [26,27].

The failure to suppress infection in the Río Cayapas might indicate the need for a change in treatment strategy. Studies conducted in Cameroon and Guatemala show that treatment every 3 months with standard doses of ivermectin is safe and well-tolerated and has significant macrofilaricidal effects against adult *O. volvulus *[28,29]. In fact, histological studies of onchocercal nodules from Ecuador from individuals with a mean of only 8.2 treatments with ivermectin have shown dramatic effects on adult female viability and fertility and also marked reductions in the frequencies of male worms [30], and provided a histological picture similar to that reported in nodules collected in the Onchocerciasis Control Program area of West Africa after 9–10 years of vector control [30]. These observations indicate that the period for which treatment must be maintained after suppression of infection in a community is achieved could be less than previously estimated if adult worms die or cannot maintain sexual reproduction earlier than previously estimated. It is possible that an intensive program of increased frequency of ivermectin treatment (i.e. 3-monthly) in combination with a course of doxycycline that can sterilize adult females worms [31] in skin test positive individuals and the treatment of repeated treatment refusers and migrants could yield rapid results in the suppression of transmission of *O. volvulus*.

The plan for certification of the elimination of onchocerciasis envisaged by OEPA comprises four phases [15]: phase I – treatment coverage of greater than 85% of the population with twice-annual ivermectin treatments for a period of 2–4 years (or 4–8 rounds of treatment) causing temporary interruption of transmission; phase II – continued suppression of *O. volvulus *with biannual high coverage treatments with no blackflies infected with *O. volvulus *and no new individuals being infected (i.e. zero incidence of infection) for the lifespan of the adult female worms (e.g. 13–14 years [21]); phase III – starting 12–14 years after the suppression of infection (or 14–18 years after the start of ivermectin treatments) when all adult females have died and suppression of infection is no longer dependent on ivermectin and consists of a 3 year 'surveillance' period of pre-certification during which ivermectin treatment is suspended; and phase IV – certification of elimination of the *O. volvulus *infection.

Our previous observations in the Río Santiago sub-focus [11] would support the time periods provided for phases I and II, but to achieve phase I in the hyperendemic area of the Río Cayapas sub-focus is likely to require much longer with the current twice-annual treatment strategy. It is likely that the periods required for each phase will vary according to the efficiency of the vector species and other factors unique to each geographic sub-focus (e.g. treatment coverage, migration patterns). Our data indicate that the Río Santiago sub-focus might be ready for the third phase of pre-certification. Pre-certification and suspension of ivermectin treatment could be considered also in Río Onzole sub-focus and satellite foci where annual or semi-annual treatments have been provided continuously for at least 13 years.

Before suspension of treatment, a survey of all endemic communities within each sub-focus should be conducted to ensure that the findings in the sentinel communities are generalizeable to non-sentinel communities. These surveys should include more sensitive diagnostic methods including the detection of *O. volvulus *DNA in skin snips [32] and specific antibodies using the OV16 immunochromatic cards [23,33] to exclude very low-level infections and histologic analysis of the viability and fertility of female worms in onchocercal nodules (if present). Detailed vectors studies might be required to examine the flight range and dispersal patterns of *Simulium *blackflies from communities in the Río Cayapas where transmission persists because of the potential risk of dispersal of infected *Simulium*. Also required will be a study of migration to determine if there are significant migrations of potentially infected individuals from the hyperendemic communities in the Río Cayapas to the Río Santiago and vice versa. Pre-certification of these foci of infection could reduce the cost of current control activities by approximately 75% (from US $250000 to just over US $60000) and release resources for an intensive elimination strategy in the Río Cayapas sub-focus.

## Conclusion

The National Program for Onchocerciasis Elimination in Ecuador has pioneered the use of community health workers to distribute ivermectin and the integration of ivermectin distribution within primary health care activities. The diversification of the activities of the control team and the integration of control within primary health care services has become increasingly important in maintaining high rates of coverage with ivermectin as the public health threat posed by onchocerciasis is perceived to be less and less important by the inhabitants of endemic communities. The data from this study provide evidence that an operational program in Ecuador might have eliminated ocular onchocerciasis as a public health problem and has made significant progress towards the goal of the elimination of the infection from Ecuador, and provides a model control program for the evaluation of the potential for the elimination of onchocerciasis from the Americas. The elimination of onchocerciasis from Latin America and Africa is an important health priority and the data from the current study provides evidence that an ivermectin-based treatment program could be a feasible strategy for the elimination of the infection from other foci of infection.

## Abbreviations

OEPA: Onchocerciasis Elimination Program for the Americas; MMFL: Mean microfilarial load; PCR: Polymerase chain reaction; DNA: Deoxyribonucleic acid

## Competing interests

The author(s) declare that they have no competing interests.

## Authors' contributions

JCV is the coordinator for the Programa Nacional de Eliminación de la Oncocercosis en el Ecuador and supervised the collection of blackflies. PJC is an unpaid technical consultant to the program, and drafted the manuscript and prepared it for publication. RL supervised the community treatments with ivermectin. TM is a former coordinator of the program and is an unpaid technical consultant to the program. JR and RP performed the ophthalmological examinations. AAL assisted in the collection of blackflies and performed the PCR analyses. RHG helped draft the manuscript. JRG was the Director of the Program until his death on 4 September 2006. All authors read and approved the final manuscript.

## Pre-publication history

The pre-publication history for this paper can be accessed here:



## Figures and Tables

**Table 1:** Characteristics of ivermectin mass distribution in the 119 comunities endemic for onchocerciasis in Ecuador

Location/endemicity	Ivermectin treatments			Mean treatment coverage % (range)
		
	No. of communities	Year of first treatment	No. treatments Median (range)	
Río Cayapas:				
Hyperendemic	31	1991–1996*	22 (13–22)	84.8 (49.9–95.3)
Mesoendemic	9	1991	16 (16–16)	86.7 (74.2–97.7)
Hypoendemic	7	1991–1993	16 (14–16)	84.7 (71.0–94.4)
Río Onzole:				
Hyperendemic	3	1991–1992	16 (15–17)	84.5 (63.4–98.1)
Mesoendemic	1	1991	16	90.3 (72.7–100)
Hypoendemic	13	1991–1993	15 (15–16)	87.2 68.5–97.2)
Río Santiago:				
Hyperendemic	8	1990–1991†	19 (17–19)	90.7 (78.1–98.7)
Mesoendemic	4	1990	18 (17–19)	87.7 (65.4–99.0)
Hypoendemic	23	1990–1991	16 (16–17)	88.9 (81.1–100)
Satelite foci:				
Mesoendemic	9	1991–1993	18 (14–19)	83.2 (37.3–97.9)
Hypoendemic	11	1990–1997	13 (11–17)	89.9 (48.7–98.4)

*In the Río Cayapas first treatments with ivermectin were provided in the hyperendemic communities in the following years: 1991 (23 communities), 1992 (1), 1995 (1), and 1996 (6). †In the Río Santiago first treatments with ivermectin were provided in the hyperendemic communities in the following years: 1990 (7 communities) and 1991 (1 community).

**Table 2:** Characteristics of the seven sentinel communities in Esmeraldas Province, Ecuador selected to evaluate the impact of ivermectin treatment on *O. volvulus *infection

Community	Location/endemicity	Ivermectin treatments				Mean treatment coverage % (range)
		
		Treatment eligible*	Annual	Twice annual	Treatments	
Corriente	Río Cayapas/Hyperendemic	173	1991- 1995	1992–1994	21	86.1 (19.1–99.4)
Grande			1997	1998–1999		
			2000	2001–2003		
El Tigre	Río Cayapas/Hyperendemic	121	1996–1997	1998–1999	13	94.4 (82.3–100)
			2000	2001–2003		
San Miguel	Río Cayapas/Hyperendemic	175	1996–1997	1998–1999	13	91.8 (85.7–97.9)
			2000	2001–2003		
Playa de	Río Santiago/Hyperendemic	236	1990	1991–1992	19	93.3 (81.8–100)
Oro			1993–2000	2001–2003		
Guayabal	Río Santiago/Hyperendemic	101	1990	1991–1992	19	84.7 (64.4–100)
			1993–2000	2001–2003		
Angostura	Río Santiago/Hyperendemic	72	1992–2000	1991	19	89.4 (75.9–100)
				2001–2003		
Naranjal	Río Canandé/Mesoendemic	491	1994–2000	1991–1993	19	83.9 (48.5–96)
				2001–2003		

*Data from 2004 census.

**Table 3:** Changes in the epidemiology of *O. volvulus *infection in the sentinel communities

Community	1989 survey		2000 survey		2004 survey	
	
	Prevalence (%)	MMFL	Prevalence (%)	MMFL	Prevalence (%)	MMFL
Corriente Grande	96.3	34.8	4.8	0.045	0.6	0.003
El Tigre	94.2	59.3	14.0	0.131	2.1	0.007
San Miguel	96.3	57.4	5.1	0.582	1.3	0.007
Playa del Oro	66.6	23.6	0	0	0	0
Guayabal	63.6	28.4	0	0	0	0
Angostura	80.6	34.7	0	0	0	0
Naranjal	53.3	21.2	0.3	0.001	0	0

The prevalence of infection and microfilarial load with *O. volvulus *in the sentinel communities is shown. Surveys were conducted in the seven sentinel communities before ivermectin treatment in 1989 and in 2000 and 2004 after the start of treatment. MMFL, Mean Microfilarial Load (geometric mean mf/mg  skin).

**Table 4:** Changes in the prevalence of ocular onchocerciasis in the sentinel communities

Sentinel community	Presence of punctate keratitis (PK, %)			Presence of microfilariae in the anterior chamber (MfAC, %)		
	
	1989	2000	2004	1989	2000	2004
Corriente Grande	41.7	1.2	0	50.5	0	0
El Tigre	36.6	1.6	0	27.3	0	0
San Miguel	50.0	4.7	0	50.0	0	0
Playa del Oro	48.8	5.1	0	6.0	0	0
Guayabal	14.3	1.5	0	0	0	0
Angostura	21.4	0	0	14.3	0	0
Naranjal	0	0.9	0	0	0	0

The prevalence of ocular onchocerciasis of the anterior segment is provided by punctate keratitis (PK) and presence of microfilariae in the anterior chamber (MfAC).

**Table 5:** Changes in infection rates with *O. volvulus *in blackflies in the sentinel communities

Sentinel community	1995		2000		2004	
	
	Blackflies analysed (N)	Infection rate	Blackflies analysed (N)	Infection rate	Blackflies analysed (N)	Infection rate
Corriente Grande	NP	NP	23500	2.1 (0.7–5.0)	12700	0
El Tigre	2550	72.7 (42.0–116.0)	17000	9.0 (4.5–16.1)	10100	6.0 (2.05–13.3)
San Miguel	3550	65.0 (39.0–101.0)	9600	1.1 (0.03–6.4)	12500	0.8 (0.02–4.1)
Playa de Oro	NP	NP	10250	0	9500	0
Guayabal	NP	NP	9950	0	10000	0
Angostura	NP	NP	9700	0	11400	0
Naranjal	NP	NP	20450	0.5 (0.01–2.8)	26050	0

Infection rates (number of flies infected per 10000 flies) with *O. volvulus *among *Simulium *blackflies collected before the start of ivermectin distribution (1995), and during the 2000 and 2004 surveys. Infection rates were calculated using Poolscreen 2.0 software. NP – not performed.
